# Supplementary figures and images for: Performance of In Silico Prediction Tools for the Detection of Germline Copy Number Variations in Cancer Predisposition Genes in 4208 Female Index Patients with Familial Breast and Ovarian Cancer
Source: Cancers (Basel). 2021 Jan 1;13(1):118. doi: 10.3390/cancers13010118 (PMC7794674; doi:10.3390/cancers13010118)

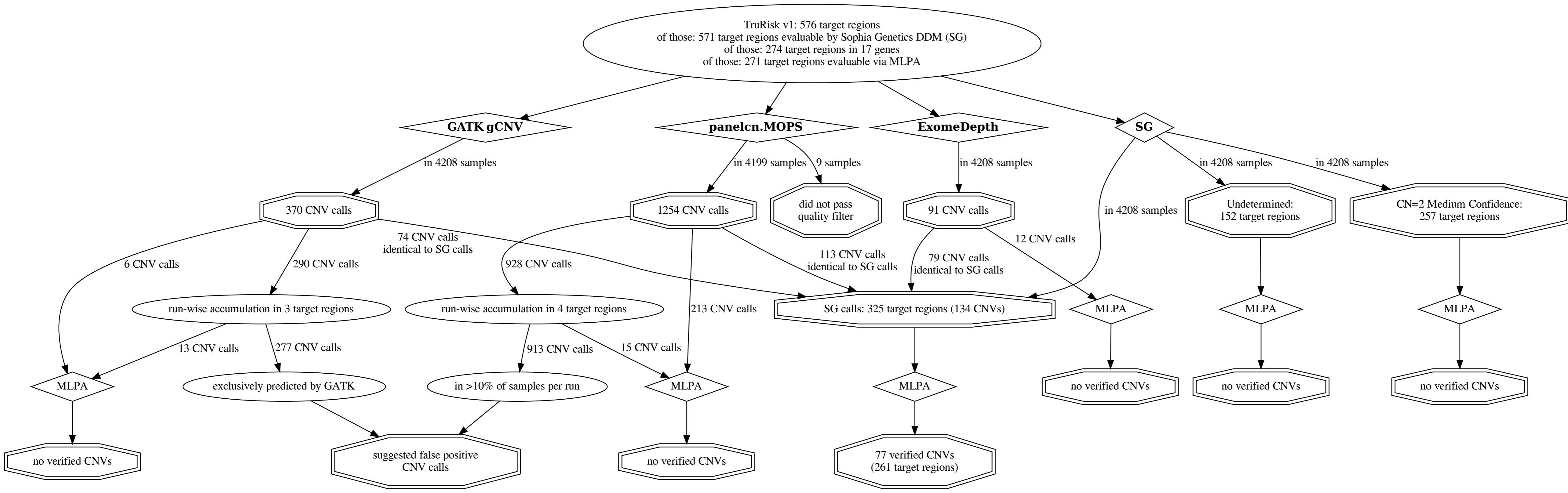

Supplement: Supplementary file 1 [file cancers-13-00118-s001.zip › FigureS1.pdf]
